# (2*E*)-3-(6-Chloro-2-meth­oxy­quinolin-3-yl)-1-(2-methyl-4-phenyl­quinolin-3-yl)prop-2-en-1-one acetone monosolvate

**DOI:** 10.1107/S1600536813020217

**Published:** 2013-07-27

**Authors:** R. Prasath, S. Sarveswari, Seik Weng Ng, Edward R. T. Tiekink

**Affiliations:** a Department of Chemistry, BITS, Pilani – K. K. Birla Goa Campus, Goa 403 726, India; b Centre for Organic and Medicinal Chemistry, VIT University, Vellore 632 014, India; c Department of Chemistry, University of Malaya, 50603 Kuala Lumpur, Malaysia; d Chemistry Department, Faculty of Science, King Abdulaziz University, PO Box 80203 Jeddah, Saudi Arabia

## Abstract

In the title solvate, C_29_H_21_ClN_2_O_2_·C_3_H_6_O, a prop-2-en-1-one bridge links two quinolinyl residues; the latter are almost perpendicular [dihedral angle = 78.27 (6)°]. The dihedral angle between the quinonyl ring system and its pendant phenyl group is 59.78 (8)°. A small twist in the bridging prop-2-en-1-one group is noted [O=C—C=C torsion angle = −10.6 (3)°]. In the crystal, a three-dimensional architecture arises as a result of C—H⋯O and π–π stacking [centroid–centroid distances = 3.5504 (12)–3.6623 (12) Å].

## Related literature

For background details and the biological applications of quinolinyl derivatives, see: Joshi *et al.* (2011 ▶); Prasath *et al.* (2013*a* ▶). For a related structure, see: Prasath *et al.* (2013*b* ▶).
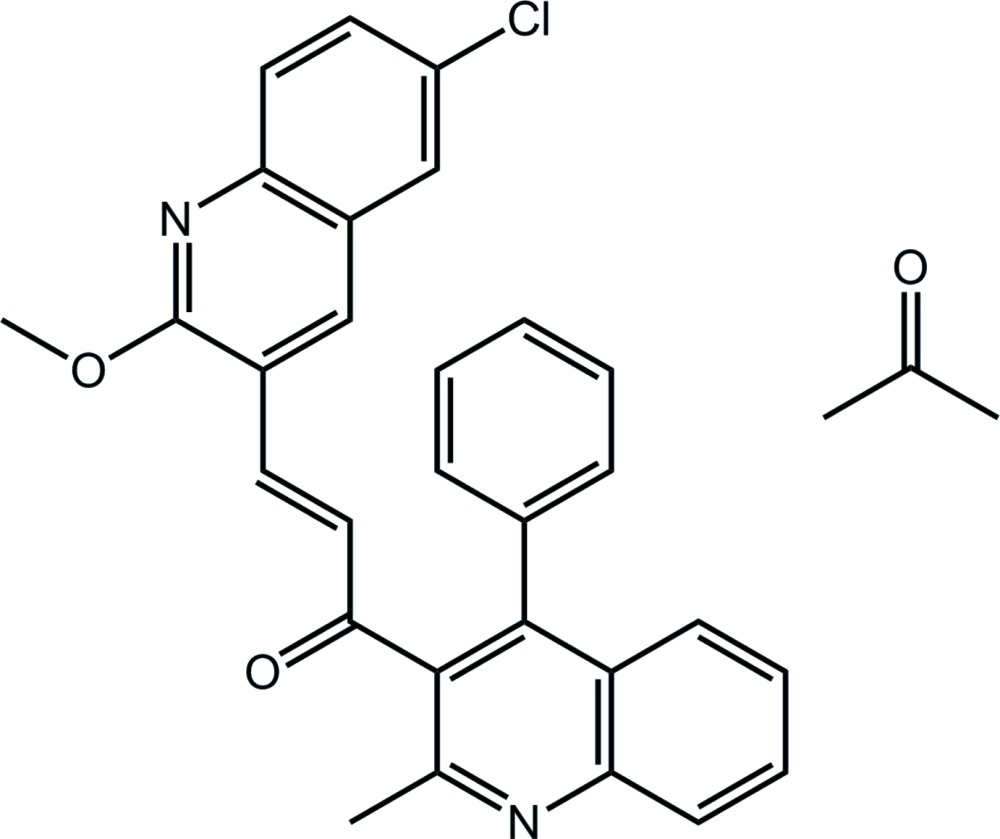


## Experimental

### 

#### Crystal data

C_29_H_21_ClN_2_O_2_·C_3_H_6_O
*M* *_r_* = 523.01
Monoclinic, 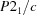

*a* = 17.1714 (3) Å
*b* = 10.7099 (2) Å
*c* = 14.5248 (2) Å
β = 100.021 (2)°
*V* = 2630.42 (8) Å^3^
*Z* = 4
Cu *K*α radiation
μ = 1.58 mm^−1^
*T* = 100 K
0.30 × 0.25 × 0.20 mm

#### Data collection

Agilent SuperNova Dual diffractometer with an Atlas detector
Absorption correction: multi-scan (*CrysAlis PRO*; Agilent, 2013 ▶) *T*_min_ = 0.665, *T*_max_ = 1.000
11367 measured reflections
5408 independent reflections
4574 reflections with *I* > 2σ(*I*)
*R*_int_ = 0.032

#### Refinement

*R*[*F* ^2^ > 2σ(*F* ^2^)] = 0.058
*wR*(*F* ^2^) = 0.167
*S* = 1.03
5408 reflections
346 parameters
H-atom parameters constrained
Δρ_max_ = 1.47 e Å^−3^
Δρ_min_ = −0.46 e Å^−3^

### 

Data collection: *CrysAlis PRO* (Agilent, 2013 ▶); cell refinement: *CrysAlis PRO*; data reduction: *CrysAlis PRO*; program(s) used to solve structure: *SHELXS97* (Sheldrick, 2008 ▶); program(s) used to refine structure: *SHELXL97* (Sheldrick, 2008 ▶); molecular graphics: *ORTEP-3 for Windows* (Farrugia, 2012 ▶) and *DIAMOND* (Brandenburg, 2006 ▶); software used to prepare material for publication: *publCIF* (Westrip, 2010 ▶).

## Supplementary Material

Crystal structure: contains datablock(s) general, I. DOI: 10.1107/S1600536813020217/hg5334sup1.cif

Structure factors: contains datablock(s) I. DOI: 10.1107/S1600536813020217/hg5334Isup2.hkl

Supplementary material file. DOI: 10.1107/S1600536813020217/hg5334Isup3.cml

Additional supplementary materials:  crystallographic information; 3D view; checkCIF report

## Figures and Tables

**Table 1:** Hydrogen-bond geometry (Å, °)

*D*—H⋯*A*	*D*—H	H⋯*A*	*D*⋯*A*	*D*—H⋯*A*
C26—H26⋯O2^i^	0.95	2.47	3.319 (3)	149
C30—H30*A*⋯O1^i^	0.98	2.52	3.373 (4)	146
C28—H28⋯O3	0.95	2.57	3.467 (3)	158

Symmetry code: (i) 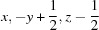.

**Fig. 1.**
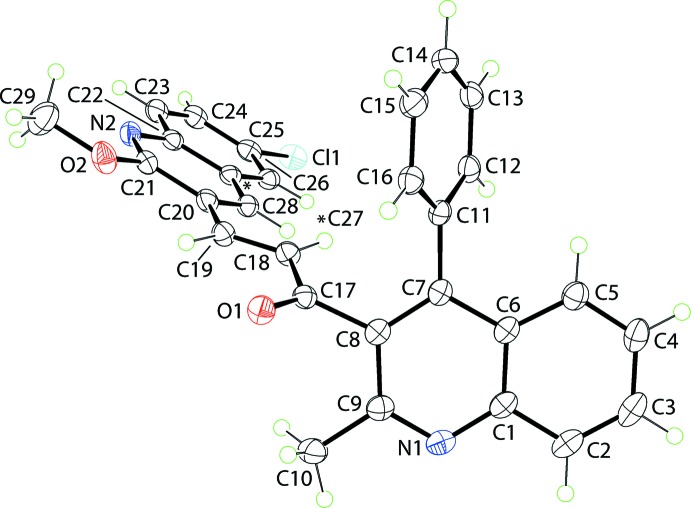
The molecular structure of (I) showing the atom-labelling scheme and displacement ellipsoids at the 50% probability level.

**Fig. 2.**
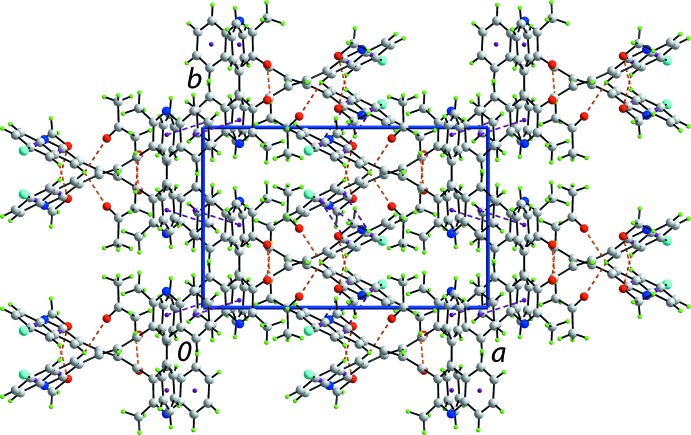
View in projection down the c axis of the unit-cell contents of (I). The π—π and C—H···O interactions are shown as purple and orange dashed lines, respectively.

## supplementary crystallographic information

### Comment

The the title compound, (I), was investigated in connection with on-going studies of quinolinyl chalcones (Prasath *et al.*, 2013*a*), motivated by their potential anti-bacterial, anti-fungal, anti-malarial and anti-cancer activity (Joshi *et al.*, 2011).

The molecular structure of the quinolinyl derivative, (I), Fig. 1, comprises two quinolinyl residues connected by the ends of a prop-2-en-1-one bridge, in an almost perpendicular relationship; the dihedral angle between the quinolinyl residues is 78.27 (6)°. The phenyl ring is inclined with respect to the quinolinyl residue to which it is attached, forming a dihedral angle of 59.78 (8)°. The conformation about the ethylene bond [C18═C19 = 1.336 (3) Å] is *E*. A small twist in the bridging prop-2-en-1-one group is manifested in the O1—C17—C18—C19 torsion angle of -10.6 (3)°. An distinct conformation was reported recently for a related structure, namely (2*E*)-3-(6-chloro-2-methoxyquinolin-3-yl)-1-(2,4-dimethylquinolin-3 - *y*)prop-2-en-1-one (Prasath *et al.*, 2013*b*) where the nitrogen atoms are approximately *syn* as opposed to approximately *anti* in (I).

In the crystal packing, the quinolinyl and acetone molecules are connected by C—H···O interactions, Table 1. Additional C—H···O contacts and a number of π—π interactions, involving pyridyl, a quinolinyl-C_6_ ring and the phenyl group, connect molecules into a three-dimensional architecture [centroid···centroid distances = 3.5504 (12), 3.5747 (12) and 3.6623 (12) Å], Fig. 2.

### Experimental

A mixture of 3-acetyl-2-methyl-4-phenylquinoline (260 mg, 0.001 *M*) and 2,6-dichloroquinoline-3-carbaldehyde (230 mg, 0.001 *M*) in methanol (20 ml) containing potassium hydroxide (0.2 g) was stirred at room temperature for 12 h. The reaction mixture was then neutralized with dilute acetic acid and the resultant solid was filtered, dried and purified by column chromatography using ethyl acetate - hexane (3:1) mixture to afford compound. Re-crystallization was by slow evaporation of an acetone solution of (I), which yielded blocks in 62% yield; *M*.pt: 366–368 K.

### Refinement

Carbon-bound H-atoms were placed in calculated positions [C—H = 0.95–0.98 Å, *U*_iso_(H) = 1.2–1.5*U*_eq_(C)] and were included in the refinement in the riding model approximation. The maximum and minimum residual electron density peaks of 1.47 and 0.46 e Å^-3^, respectively, were located 0.85 Å and 0.68 Å from the O2 and Cl1 atoms, respectively.

### Crystal data

**Table:**

C_29_H_21_ClN_2_O_2_·C_3_H_6_O	*F*(000) = 1096
*M**_r_* = 523.01	*D*_x_ = 1.321 Mg m^−^^3^
Monoclinic, *P*2_1_/*c*	Cu *K*α radiation, λ = 1.54184 Å
Hall symbol: -P 2ybc	Cell parameters from 4229 reflections
*a* = 17.1714 (3) Å	θ = 2.6–76.5°
*b* = 10.7099 (2) Å	µ = 1.58 mm^−^^1^
*c* = 14.5248 (2) Å	*T* = 100 K
β = 100.021 (2)°	Prism, pale-yellow
*V* = 2630.42 (8) Å^3^	0.30 × 0.25 × 0.20 mm
*Z* = 4	

### Data collection

**Table:**

Agilent SuperNova Dual diffractometer with an Atlas detector	5408 independent reflections
Radiation source: SuperNova (Cu) X-ray Source	4574 reflections with *I* > 2σ(*I*)
Mirror monochromator	*R*_int_ = 0.032
Detector resolution: 10.4041 pixels mm^-1^	θ_max_ = 76.7°, θ_min_ = 2.6°
ω scan	*h* = −21→21
Absorption correction: multi-scan (*CrysAlis PRO*; Agilent, 2013)	*k* = −9→13
*T*_min_ = 0.665, *T*_max_ = 1.000	*l* = −18→11
11367 measured reflections	

### Refinement

**Table:**

Refinement on *F*^2^	Primary atom site location: structure-invariant direct methods
Least-squares matrix: full	Secondary atom site location: difference Fourier map
*R*[*F*^2^ > 2σ(*F*^2^)] = 0.058	Hydrogen site location: inferred from neighbouring sites
*wR*(*F*^2^) = 0.167	H-atom parameters constrained
*S* = 1.03	*w* = 1/[σ^2^(*F*_o_^2^) + (0.0953*P*)^2^ + 1.726*P*] where *P* = (*F*_o_^2^ + 2*F*_c_^2^)/3
5408 reflections	(Δ/σ)_max_ < 0.001
346 parameters	Δρ_max_ = 1.47 e Å^−^^3^
0 restraints	Δρ_min_ = −0.46 e Å^−^^3^

### Special details

**Table:**

Geometry. All s.u.'s (except the s.u. in the dihedral angle between two l.s. planes) are estimated using the full covariance matrix. The cell s.u.'s are taken into account individually in the estimation of s.u.'s in distances, angles and torsion angles; correlations between s.u.'s in cell parameters are only used when they are defined by crystal symmetry. An approximate (isotropic) treatment of cell s.u.'s is used for estimating s.u.'s involving l.s. planes.
Refinement. Refinement of *F*^2^ against ALL reflections. The weighted *R*-factor *wR* and goodness of fit *S* are based on *F*^2^, conventional *R*-factors *R* are based on *F*, with *F* set to zero for negative *F*^2^. The threshold expression of *F*^2^ > 2σ(*F*^2^) is used only for calculating *R*-factors(gt) *etc*. and is not relevant to the choice of reflections for refinement. *R*-factors based on *F*^2^ are statistically about twice as large as those based on *F*, and *R*- factors based on ALL data will be even larger.

### Fractional atomic coordinates and isotropic or equivalent isotropic displacement parameters (Å^2^)

**Table:**

	*x*	*y*	*z*	*U*_iso_*/*U*_eq_	
Cl1	0.62921 (3)	0.12909 (5)	0.32316 (4)	0.03232 (17)	
O1	0.22537 (9)	0.34889 (15)	0.74016 (10)	0.0264 (3)	
O2	0.47465 (9)	0.10414 (17)	0.80988 (11)	0.0324 (4)	
N1	0.12879 (10)	0.59002 (17)	0.50819 (13)	0.0258 (4)	
N2	0.55712 (10)	0.06750 (17)	0.70334 (12)	0.0245 (4)	
C1	0.08058 (12)	0.5228 (2)	0.44105 (14)	0.0235 (4)	
C2	0.03155 (13)	0.5910 (2)	0.36971 (15)	0.0295 (5)	
H2	0.0332	0.6797	0.3702	0.035*	
C3	−0.01806 (14)	0.5301 (2)	0.30037 (15)	0.0338 (5)	
H3	−0.0501	0.5767	0.2525	0.041*	
C4	−0.02195 (13)	0.3989 (2)	0.29950 (15)	0.0314 (5)	
H4	−0.0567	0.3575	0.2510	0.038*	
C5	0.02415 (12)	0.3303 (2)	0.36832 (14)	0.0264 (4)	
H5	0.0204	0.2418	0.3677	0.032*	
C6	0.07738 (11)	0.3911 (2)	0.44037 (13)	0.0216 (4)	
C7	0.12817 (11)	0.32582 (19)	0.51344 (13)	0.0201 (4)	
C8	0.17507 (11)	0.39553 (19)	0.58093 (13)	0.0210 (4)	
C9	0.17372 (12)	0.5289 (2)	0.57624 (14)	0.0241 (4)	
C10	0.22557 (14)	0.6057 (2)	0.64860 (17)	0.0332 (5)	
H10A	0.2229	0.6936	0.6293	0.050*	
H10B	0.2803	0.5763	0.6551	0.050*	
H10C	0.2074	0.5976	0.7087	0.050*	
C11	0.12677 (11)	0.18704 (19)	0.51703 (13)	0.0211 (4)	
C12	0.14436 (12)	0.1150 (2)	0.44272 (14)	0.0242 (4)	
H12	0.1597	0.1550	0.3903	0.029*	
C13	0.13946 (13)	−0.0141 (2)	0.44520 (16)	0.0293 (5)	
H13	0.1517	−0.0619	0.3946	0.035*	
C14	0.11663 (13)	−0.0742 (2)	0.52142 (17)	0.0317 (5)	
H14	0.1125	−0.1627	0.5225	0.038*	
C15	0.10015 (13)	−0.0036 (2)	0.59537 (16)	0.0305 (5)	
H15	0.0852	−0.0440	0.6479	0.037*	
C16	0.10515 (12)	0.1257 (2)	0.59373 (14)	0.0250 (4)	
H16	0.0938	0.1729	0.6452	0.030*	
C17	0.23238 (11)	0.33553 (18)	0.65850 (13)	0.0208 (4)	
C18	0.29959 (11)	0.27093 (19)	0.62832 (13)	0.0217 (4)	
H18	0.2965	0.2518	0.5639	0.026*	
C19	0.36459 (11)	0.23825 (19)	0.68814 (14)	0.0223 (4)	
H19	0.3649	0.2491	0.7531	0.027*	
C20	0.43552 (11)	0.18660 (19)	0.65883 (13)	0.0218 (4)	
C21	0.49258 (12)	0.11715 (19)	0.72277 (14)	0.0231 (4)	
C22	0.57217 (11)	0.08240 (19)	0.61423 (14)	0.0221 (4)	
C23	0.64121 (12)	0.0285 (2)	0.59056 (15)	0.0266 (4)	
H23	0.6762	−0.0175	0.6360	0.032*	
C24	0.65784 (12)	0.0425 (2)	0.50195 (16)	0.0273 (4)	
H24	0.7041	0.0059	0.4861	0.033*	
C25	0.60641 (12)	0.1109 (2)	0.43496 (15)	0.0244 (4)	
C26	0.53820 (12)	0.16319 (19)	0.45448 (14)	0.0229 (4)	
H26	0.5035	0.2075	0.4076	0.027*	
C27	0.52045 (11)	0.15016 (18)	0.54522 (14)	0.0204 (4)	
C28	0.45154 (11)	0.20257 (19)	0.57037 (14)	0.0215 (4)	
H28	0.4161	0.2491	0.5257	0.026*	
C29	0.52882 (18)	0.0347 (3)	0.87429 (17)	0.0424 (6)	
H29A	0.5101	0.0305	0.9342	0.064*	
H29B	0.5807	0.0753	0.8832	0.064*	
H29C	0.5332	−0.0500	0.8501	0.064*	
O3	0.33767 (14)	0.4389 (2)	0.45197 (14)	0.0573 (6)	
C30	0.23862 (15)	0.4489 (3)	0.31684 (17)	0.0373 (6)	
H30A	0.2371	0.3579	0.3235	0.056*	
H30B	0.2551	0.4700	0.2574	0.056*	
H30C	0.1859	0.4835	0.3178	0.056*	
C31	0.29599 (14)	0.5025 (2)	0.39548 (15)	0.0331 (5)	
C32	0.29821 (18)	0.6428 (3)	0.40126 (19)	0.0450 (6)	
H32A	0.3467	0.6692	0.4427	0.068*	
H32B	0.2521	0.6728	0.4262	0.068*	
H32C	0.2973	0.6777	0.3387	0.068*	

### Atomic displacement parameters (Å^2^)

**Table:**

	*U*^11^	*U*^22^	*U*^33^	*U*^12^	*U*^13^	*U*^23^
Cl1	0.0329 (3)	0.0336 (3)	0.0345 (3)	−0.0039 (2)	0.0173 (2)	−0.0023 (2)
O1	0.0261 (7)	0.0297 (8)	0.0242 (7)	0.0027 (6)	0.0063 (6)	−0.0013 (6)
O2	0.0278 (8)	0.0422 (10)	0.0265 (7)	0.0063 (7)	0.0026 (6)	−0.0116 (7)
N1	0.0244 (9)	0.0217 (9)	0.0326 (9)	0.0024 (7)	0.0087 (7)	0.0035 (7)
N2	0.0223 (8)	0.0263 (9)	0.0231 (8)	0.0029 (7)	−0.0008 (6)	−0.0031 (7)
C1	0.0213 (9)	0.0268 (11)	0.0245 (9)	0.0063 (8)	0.0098 (8)	0.0045 (8)
C2	0.0300 (11)	0.0297 (11)	0.0310 (11)	0.0103 (9)	0.0111 (9)	0.0089 (9)
C3	0.0296 (11)	0.0444 (14)	0.0279 (11)	0.0145 (10)	0.0063 (9)	0.0108 (10)
C4	0.0242 (10)	0.0431 (14)	0.0261 (10)	0.0082 (9)	0.0015 (8)	0.0005 (9)
C5	0.0235 (10)	0.0299 (11)	0.0261 (10)	0.0042 (8)	0.0054 (8)	0.0003 (8)
C6	0.0179 (9)	0.0264 (10)	0.0219 (9)	0.0040 (7)	0.0077 (7)	0.0031 (8)
C7	0.0148 (8)	0.0252 (10)	0.0216 (9)	0.0019 (7)	0.0064 (7)	0.0016 (7)
C8	0.0174 (9)	0.0247 (10)	0.0223 (9)	0.0019 (7)	0.0076 (7)	0.0015 (7)
C9	0.0201 (9)	0.0241 (10)	0.0294 (10)	0.0019 (8)	0.0077 (8)	0.0000 (8)
C10	0.0317 (11)	0.0225 (11)	0.0431 (13)	−0.0013 (9)	−0.0003 (10)	−0.0022 (9)
C11	0.0166 (8)	0.0224 (10)	0.0236 (9)	0.0024 (7)	0.0014 (7)	0.0009 (7)
C12	0.0195 (9)	0.0292 (11)	0.0229 (9)	0.0028 (8)	0.0008 (7)	−0.0024 (8)
C13	0.0229 (10)	0.0287 (11)	0.0330 (11)	0.0050 (8)	−0.0046 (8)	−0.0090 (9)
C14	0.0270 (10)	0.0205 (10)	0.0433 (12)	0.0009 (8)	−0.0062 (9)	−0.0008 (9)
C15	0.0251 (10)	0.0287 (11)	0.0358 (11)	−0.0027 (8)	0.0005 (9)	0.0065 (9)
C16	0.0218 (9)	0.0275 (11)	0.0250 (10)	−0.0002 (8)	0.0025 (7)	0.0014 (8)
C17	0.0184 (9)	0.0201 (9)	0.0238 (9)	−0.0013 (7)	0.0035 (7)	−0.0010 (7)
C18	0.0199 (9)	0.0231 (10)	0.0227 (9)	0.0012 (7)	0.0052 (7)	−0.0035 (7)
C19	0.0209 (9)	0.0242 (10)	0.0217 (9)	0.0002 (7)	0.0034 (7)	−0.0025 (7)
C20	0.0178 (9)	0.0225 (10)	0.0242 (9)	−0.0005 (7)	0.0011 (7)	−0.0042 (8)
C21	0.0247 (10)	0.0232 (10)	0.0202 (9)	−0.0008 (8)	0.0009 (7)	−0.0032 (7)
C22	0.0185 (9)	0.0192 (9)	0.0276 (10)	−0.0018 (7)	0.0007 (7)	−0.0035 (8)
C23	0.0177 (9)	0.0264 (10)	0.0341 (11)	0.0010 (8)	−0.0001 (8)	−0.0045 (9)
C24	0.0174 (9)	0.0273 (11)	0.0371 (11)	−0.0012 (8)	0.0047 (8)	−0.0076 (9)
C25	0.0218 (9)	0.0240 (10)	0.0294 (10)	−0.0055 (8)	0.0097 (8)	−0.0040 (8)
C26	0.0219 (9)	0.0199 (10)	0.0268 (10)	−0.0022 (7)	0.0039 (7)	−0.0002 (8)
C27	0.0169 (9)	0.0179 (9)	0.0261 (9)	−0.0012 (7)	0.0028 (7)	−0.0021 (7)
C28	0.0175 (9)	0.0208 (9)	0.0255 (9)	−0.0006 (7)	0.0019 (7)	−0.0015 (7)
C29	0.0550 (16)	0.0437 (15)	0.0290 (12)	0.0158 (12)	0.0087 (11)	0.0058 (10)
O3	0.0579 (13)	0.0665 (15)	0.0453 (11)	0.0049 (11)	0.0028 (10)	0.0181 (10)
C30	0.0363 (12)	0.0427 (14)	0.0359 (12)	−0.0130 (10)	0.0146 (10)	−0.0117 (10)
C31	0.0324 (12)	0.0415 (14)	0.0269 (10)	−0.0060 (10)	0.0098 (9)	0.0035 (9)
C32	0.0560 (17)	0.0404 (15)	0.0407 (13)	−0.0156 (12)	0.0142 (12)	−0.0066 (11)

### Geometric parameters (Å, º)

**Table:**

Cl1—C25	1.746 (2)	C14—H14	0.9500
O1—C17	1.221 (2)	C15—C16	1.388 (3)
O2—C21	1.360 (3)	C15—H15	0.9500
O2—C29	1.411 (3)	C16—H16	0.9500
N1—C9	1.317 (3)	C17—C18	1.476 (3)
N1—C1	1.369 (3)	C18—C19	1.336 (3)
N2—C21	1.304 (3)	C18—H18	0.9500
N2—C22	1.373 (3)	C19—C20	1.467 (3)
C1—C6	1.411 (3)	C19—H19	0.9500
C1—C2	1.419 (3)	C20—C28	1.371 (3)
C2—C3	1.366 (3)	C20—C21	1.435 (3)
C2—H2	0.9500	C22—C23	1.414 (3)
C3—C4	1.407 (4)	C22—C27	1.418 (3)
C3—H3	0.9500	C23—C24	1.374 (3)
C4—C5	1.375 (3)	C23—H23	0.9500
C4—H4	0.9500	C24—C25	1.401 (3)
C5—C6	1.422 (3)	C24—H24	0.9500
C5—H5	0.9500	C25—C26	1.372 (3)
C6—C7	1.433 (3)	C26—C27	1.410 (3)
C7—C8	1.375 (3)	C26—H26	0.9500
C7—C11	1.488 (3)	C27—C28	1.414 (3)
C8—C9	1.431 (3)	C28—H28	0.9500
C8—C17	1.505 (3)	C29—H29A	0.9800
C9—C10	1.499 (3)	C29—H29B	0.9800
C10—H10A	0.9800	C29—H29C	0.9800
C10—H10B	0.9800	O3—C31	1.202 (3)
C10—H10C	0.9800	C30—C31	1.488 (3)
C11—C16	1.398 (3)	C30—H30A	0.9800
C11—C12	1.401 (3)	C30—H30B	0.9800
C12—C13	1.386 (3)	C30—H30C	0.9800
C12—H12	0.9500	C31—C32	1.505 (4)
C13—C14	1.395 (3)	C32—H32A	0.9800
C13—H13	0.9500	C32—H32B	0.9800
C14—C15	1.383 (3)	C32—H32C	0.9800
			
C21—O2—C29	116.16 (17)	C18—C17—C8	114.89 (16)
C9—N1—C1	118.42 (19)	C19—C18—C17	122.50 (18)
C21—N2—C22	117.61 (17)	C19—C18—H18	118.7
N1—C1—C6	123.29 (18)	C17—C18—H18	118.7
N1—C1—C2	117.2 (2)	C18—C19—C20	123.50 (18)
C6—C1—C2	119.5 (2)	C18—C19—H19	118.3
C3—C2—C1	120.5 (2)	C20—C19—H19	118.3
C3—C2—H2	119.8	C28—C20—C21	116.46 (18)
C1—C2—H2	119.8	C28—C20—C19	122.50 (18)
C2—C3—C4	120.4 (2)	C21—C20—C19	121.03 (18)
C2—C3—H3	119.8	N2—C21—O2	119.87 (18)
C4—C3—H3	119.8	N2—C21—C20	125.56 (18)
C5—C4—C3	120.5 (2)	O2—C21—C20	114.56 (18)
C5—C4—H4	119.8	N2—C22—C23	119.04 (18)
C3—C4—H4	119.8	N2—C22—C27	121.91 (18)
C4—C5—C6	120.3 (2)	C23—C22—C27	119.06 (19)
C4—C5—H5	119.8	C24—C23—C22	120.2 (2)
C6—C5—H5	119.8	C24—C23—H23	119.9
C1—C6—C5	118.83 (18)	C22—C23—H23	119.9
C1—C6—C7	117.66 (18)	C23—C24—C25	119.92 (19)
C5—C6—C7	123.51 (19)	C23—C24—H24	120.0
C8—C7—C6	117.93 (19)	C25—C24—H24	120.0
C8—C7—C11	121.90 (17)	C26—C25—C24	121.83 (19)
C6—C7—C11	120.12 (17)	C26—C25—Cl1	118.97 (17)
C7—C8—C9	120.37 (18)	C24—C25—Cl1	119.20 (16)
C7—C8—C17	121.82 (18)	C25—C26—C27	118.94 (19)
C9—C8—C17	117.72 (18)	C25—C26—H26	120.5
N1—C9—C8	122.29 (19)	C27—C26—H26	120.5
N1—C9—C10	116.9 (2)	C26—C27—C28	121.92 (18)
C8—C9—C10	120.79 (19)	C26—C27—C22	120.04 (18)
C9—C10—H10A	109.5	C28—C27—C22	118.04 (18)
C9—C10—H10B	109.5	C20—C28—C27	120.41 (18)
H10A—C10—H10B	109.5	C20—C28—H28	119.8
C9—C10—H10C	109.5	C27—C28—H28	119.8
H10A—C10—H10C	109.5	O2—C29—H29A	109.5
H10B—C10—H10C	109.5	O2—C29—H29B	109.5
C16—C11—C12	118.5 (2)	H29A—C29—H29B	109.5
C16—C11—C7	120.37 (18)	O2—C29—H29C	109.5
C12—C11—C7	121.04 (18)	H29A—C29—H29C	109.5
C13—C12—C11	120.4 (2)	H29B—C29—H29C	109.5
C13—C12—H12	119.8	C31—C30—H30A	109.5
C11—C12—H12	119.8	C31—C30—H30B	109.5
C12—C13—C14	120.5 (2)	H30A—C30—H30B	109.5
C12—C13—H13	119.7	C31—C30—H30C	109.5
C14—C13—H13	119.7	H30A—C30—H30C	109.5
C15—C14—C13	119.2 (2)	H30B—C30—H30C	109.5
C15—C14—H14	120.4	O3—C31—C30	122.8 (3)
C13—C14—H14	120.4	O3—C31—C32	121.4 (2)
C14—C15—C16	120.7 (2)	C30—C31—C32	115.8 (2)
C14—C15—H15	119.6	C31—C32—H32A	109.5
C16—C15—H15	119.6	C31—C32—H32B	109.5
C15—C16—C11	120.6 (2)	H32A—C32—H32B	109.5
C15—C16—H16	119.7	C31—C32—H32C	109.5
C11—C16—H16	119.7	H32A—C32—H32C	109.5
O1—C17—C18	123.90 (18)	H32B—C32—H32C	109.5
O1—C17—C8	120.99 (18)		
			
C9—N1—C1—C6	−0.9 (3)	C7—C11—C16—C15	176.94 (18)
C9—N1—C1—C2	178.66 (18)	C7—C8—C17—O1	−117.8 (2)
N1—C1—C2—C3	179.97 (19)	C9—C8—C17—O1	65.6 (3)
C6—C1—C2—C3	−0.5 (3)	C7—C8—C17—C18	67.3 (2)
C1—C2—C3—C4	0.8 (3)	C9—C8—C17—C18	−109.3 (2)
C2—C3—C4—C5	−0.1 (3)	O1—C17—C18—C19	−10.6 (3)
C3—C4—C5—C6	−1.1 (3)	C8—C17—C18—C19	164.11 (19)
N1—C1—C6—C5	178.86 (18)	C17—C18—C19—C20	−173.03 (19)
C2—C1—C6—C5	−0.7 (3)	C18—C19—C20—C28	20.8 (3)
N1—C1—C6—C7	−0.8 (3)	C18—C19—C20—C21	−159.9 (2)
C2—C1—C6—C7	179.69 (18)	C22—N2—C21—O2	179.46 (18)
C4—C5—C6—C1	1.5 (3)	C22—N2—C21—C20	−0.1 (3)
C4—C5—C6—C7	−178.94 (19)	C29—O2—C21—N2	−0.5 (3)
C1—C6—C7—C8	1.8 (3)	C29—O2—C21—C20	179.0 (2)
C5—C6—C7—C8	−177.81 (18)	C28—C20—C21—N2	−0.8 (3)
C1—C6—C7—C11	179.30 (17)	C19—C20—C21—N2	179.8 (2)
C5—C6—C7—C11	−0.3 (3)	C28—C20—C21—O2	179.62 (18)
C6—C7—C8—C9	−1.3 (3)	C19—C20—C21—O2	0.3 (3)
C11—C7—C8—C9	−178.73 (17)	C21—N2—C22—C23	−179.30 (19)
C6—C7—C8—C17	−177.82 (17)	C21—N2—C22—C27	0.6 (3)
C11—C7—C8—C17	4.7 (3)	N2—C22—C23—C24	−179.71 (19)
C1—N1—C9—C8	1.5 (3)	C27—C22—C23—C24	0.3 (3)
C1—N1—C9—C10	−179.85 (19)	C22—C23—C24—C25	0.3 (3)
C7—C8—C9—N1	−0.4 (3)	C23—C24—C25—C26	−1.3 (3)
C17—C8—C9—N1	176.27 (18)	C23—C24—C25—Cl1	179.27 (16)
C7—C8—C9—C10	−179.01 (19)	C24—C25—C26—C27	1.6 (3)
C17—C8—C9—C10	−2.3 (3)	Cl1—C25—C26—C27	−179.00 (15)
C8—C7—C11—C16	59.1 (3)	C25—C26—C27—C28	179.44 (18)
C6—C7—C11—C16	−118.3 (2)	C25—C26—C27—C22	−0.9 (3)
C8—C7—C11—C12	−123.1 (2)	N2—C22—C27—C26	179.99 (18)
C6—C7—C11—C12	59.5 (3)	C23—C22—C27—C26	−0.1 (3)
C16—C11—C12—C13	0.7 (3)	N2—C22—C27—C28	−0.3 (3)
C7—C11—C12—C13	−177.16 (18)	C23—C22—C27—C28	179.64 (18)
C11—C12—C13—C14	0.3 (3)	C21—C20—C28—C27	1.1 (3)
C12—C13—C14—C15	−1.0 (3)	C19—C20—C28—C27	−179.53 (18)
C13—C14—C15—C16	0.8 (3)	C26—C27—C28—C20	179.06 (19)
C14—C15—C16—C11	0.2 (3)	C22—C27—C28—C20	−0.6 (3)
C12—C11—C16—C15	−0.9 (3)		

### Hydrogen-bond geometry (Å, º)

**Table:**

*D*—H···*A*	*D*—H	H···*A*	*D*···*A*	*D*—H···*A*
C26—H26···O2^i^	0.95	2.47	3.319 (3)	149
C30—H30*A*···O1^i^	0.98	2.52	3.373 (4)	146
C28—H28···O3	0.95	2.57	3.467 (3)	158

Symmetry code: (i) *x*, −*y*+1/2, *z*−1/2.
